# Proteomic analysis of heart failure hospitalization among patients with chronic kidney disease: The Heart and Soul Study

**DOI:** 10.1371/journal.pone.0208042

**Published:** 2018-12-17

**Authors:** Ruth F. Dubin, Mary Whooley, Alexander Pico, Peter Ganz, Nelson B. Schiller, Craig Meyer

**Affiliations:** 1 Division of Nephrology, San Francisco VA Medical Center/University of California, San Francisco, California, United States of America; 2 Department of Medicine, University of California San Francisco/ San Francisco VA Medical Center, San Francisco, California, United States of America; 3 University of California San Francisco Gladstone Institute, San Francisco, California, United States of America; 4 Division of Cardiology, Zuckerberg San Francisco General Hospital/University of California, San Francisco, California, United States of America; 5 Division of Cardiology, University of California San Francisco, San Francisco, California, United States of America; International University of Health and Welfare, School of Medicine, JAPAN

## Abstract

**Background:**

Patients with chronic kidney disease (CKD) are at increased risk for heart failure (HF). We aimed to investigate differences in proteins associated with HF hospitalizations among patients with and without CKD in the Heart and Soul Study.

**Methods and results:**

We measured 1068 unique plasma proteins from baseline samples of 974 participants in The Heart and Soul Study who were followed for HF hospitalization over a median of 7 years. We sequentially applied forest regression and Cox survival analyses to select prognostic proteins. Among participants with CKD, four proteins were associated with HF at Bonferroni-level significance (p<2.5x10^-4^): Angiopoietin-2 (HR[95%CI] 1.45[1.33, 1.59]), Spondin-1 (HR[95%CI] 1.13 [1.06, 1.20]), tartrate-resistant acid phosphatase type 5 (HR[95%CI] 0.65[0.53, 0.78]) and neurogenis locus notch homolog protein 1 (NOTCH1) (HR[95%CI] 0.67[0.55, 0.80]). These associations persisted at p<0.01 after adjustment for age, estimated glomerular filtration and history of HF. CKD was a significant interaction term in the associations of NOTCH1 and Spondin-1 with HF. Pathway analysis showed a trend for higher representation of the Cardiac Hypertrophy and Complement/Coagulation pathways among proteins prognostic of HF in the CKD sub-group.

**Conclusions:**

These results suggest that markers of heart failure differ between patients with and without CKD. Further research is needed to validate novel markers in cohorts of patients with CKD and adjudicated HF events.

## Introduction

The epidemic of chronic kidney disease (CKD) (estimated glomerular filtration rate (eGFR) <60 ml/min/m^2^ or albuminuria>30mg/g) affects 20 million Americans.[1] Epidemiological studies of both community-based[2–4] and high-risk populations[5–8] have confirmed that CKD confers at least a two-fold increase in cardiovascular (CV) risk.[9] Patients with mild to moderate CKD (eGFR 30–60 ml/min/m^2^) are at higher risk for dying of CV disease than progressing to end-stage renal disease.[7, 10] Albuminuria, itself sufficient to diagnose CKD (according to Kidney Disease Improving Global Outcomes Consortium (KDIGO))[11] is an independent risk factor for heart failure (HF) and myocardial infarction.[2, 12, 13]

HF is the most common CV event in the Chronic Renal Insufficiency Cohort (CRIC),[14, 15] in which the annual rates of HF, myocardial infarction and CV mortality are 1.5%, 0.8% and 1.2%, respectively. Even in patients without CKD, small decrements in renal function (measured by cystatin C), are associated with higher risk of incident HF.[4] Dysfunctional pathways contributing to this high risk of HF putatively include sympathetic over-activity leading to renin-angiotensin activation[16], and in turn, volume overload;[17] inflammation and volume overload leading to cardiac remodeling;[18] and inflammation,[19] oxidative stress and higher levels of asymmetric dimethylarginine[20] leading to endothelial dysfunction.[21] Unfortunately, we have few therapeutic targets within these pathways.

We sought to examine circulating proteins associated with HF among patients with CKD and to discern differences in biological pathways leading to HF between patients with and without CKD in the Heart and Soul Study.

## Materials and methods

All participants provided written informed consent to participate in the Heart and Soul Study. In these analyses, we included 974 participants with assessment of proteomics at baseline. Veterans Affairs Institutional Review Boards at each site approved the study protocol. All records were anonymized before statistical analysis.

### Protein selection

Proteomics was measured in baseline samples by SomaLogic (Boulder, CO) using the SOMAscan aptamer-based proteomic assay as previously described.[22] Aptamers are short single stranded RNA or DNA oligomers, synthesized as a core of 20–80 randomly selected nucleotide bases flanked by constant base sequences that may be enzymatically modified.[23] Each aptamer has a unique three-dimensional shape and binds to a surface epitope of a non-denatured protein.[24] Specificity of aptamer-protein pairs has been examined with methods including high performance liquid chromatography, mass spectrometry, and western blot.[25] The final list of 1068 proteins that we utilized in the current analyses was based on the SOMAscan version used in the previous Heart and Soul analysis,[22] with the following exceptions. We did not use quality control criteria to exclude any proteins; we did choose to include only proteins labeled as ‘human’ in the dataset supplied by Heart and Soul investigators. (See **S1 Table** for a list of proteins excluded from the analysis).

### Short statistical methods

**Fig 1:**
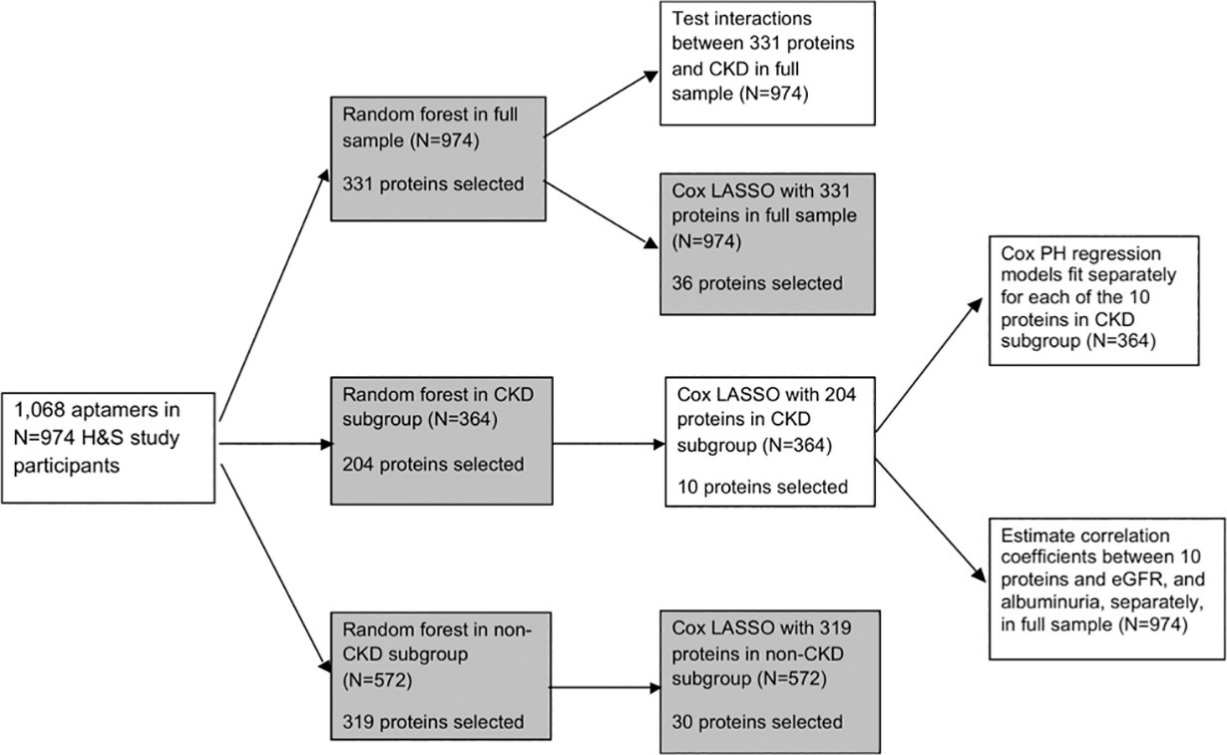
Analytic plan (See attached file). This flowchart depicts our analytic plan. Results of analyses in boxes shaded grey were performed but had lower relevance to the project Aims and thus are not included in the manuscript. H&S: Heart and Soul. LASSO: Least Absolute Shrinkage and Selection Operator. PH: proportional hazard.

First, we examined baseline characteristics of the demographics and comorbidities among the whole cohort, and groups with or without CKD. Categorical variables were evaluated by chi^2^. For continuous variables, normality was evaluated by visual inspection, equal variance by F-tests, and then Student’s t-tests, Welch’s t-tests, and Mann-Whitney tests were used as appropriate to evaluate differences in characteristics by CKD-status. We selected 1068 aptamers that were designated “human” and evaluated these as potential predictors in the random survival forests. After normal standardization of the 1068 proteins, random survival forests[26] were used to model time to HF hospitalization. Random forest regression models have several advantages, including better integration of correlated variables, excellent predictive qualities[27, 28] and the capacity to incorporate interactions between variables and non-linear relationships in the model.[29] The candidate proteins selected in the CKD-subgroup were further reduced by fitting a Cox Least Absolute Shrinkage and Selection Operator (LASSO) regression model predicting heart failure in those with CKD. After eliminating less predictive proteins by the Cox LASSO method, we fit separate Cox proportional hazards regression models for each of the final LASSO selected proteins predicting time to HF hospitalization in the CKD subgroup, successively adjusting for baseline measures of age (continuous, years), eGFR (continuous, ml/min/1.73m^2^) and history of heart failure (yes / no). To verify our LASSO analysis, we performed a sensitivity analysis. We repeated the Cox LASSO regression analyses in the CKD-subgroup instead using the set of proteins selected in the random forest for the full sample as potential predictors of heart failure hospitalization. Pearson’s correlation coefficients were estimated between proteins selected in the CKD subgroup and eGFR or albuminuria in the full sample of participants. Additionally, we tested interactions between the top predictive proteins selected in random survival forest model for the full sample and CKD (yes / no), with significance threshold for the interaction term at p<0.05. Data management and statistical analyses were conducted using R version 3.3.0.[30] Pathway analyses were performed using Gene Ontology (geneontology.org) and WikiPathways (wikipathways.org). An organizational chart for these analyses is shown in **Fig 1 (below).** A full description of the Heart and Soul Study, statistical methods and pathway analyses is found in **S1 File, Expanded Methods**.

## Results

### Baseline characteristics

Among all 974 participants for whom proteomic data were available, mean age was 67 years, 18% were women, 17% had a history of HF and 54% had a history of myocardial infarction. The majority of participants (>80%) had ejection fraction >50% and either normal or mildly impaired diastolic dysfunction. Over 1/3 of the participants had CKD (defined as eGFR<60 or albuminuria>30mg/g); these participants were older and more likely to have a history of hypertension, diabetes, MI or HF, or abnormal diastolic function **(Table 1).**

**Table 1:** Baseline patient characteristics in total sample and in sub-groups stratified on chronic kidney disease.

	Total	CKD	No CKD	p-value*
*n*	974	364	572	
**Demographic factors**				
Age (years)	66.8 (11.0)	70.8 (11.1)	64.3 (10.2)	<0.001
Male sex	797 (82%)	300 (82%)	469 (82%)	0.94
White race	587 (60%)	223 (61%)	345 (60%)	0.83
**Clinical history**				
Hypertension	689 (71%)	281 (77%)	382 (67%)	<0.001
Revascularization	571 (59%)	231 (64%)	325 (57%)	0.04
Diabetes	253 (26%)	132 (36%)	112 (20%)	<0.001
History of myocardial infarction	523 (54%)	218 (60%)	284 (50%)	0.003
History of heart failure	169 (17%)	91 (25%)	69 (12%)	<0.001
Stroke	136 (14%)	61 (17%)	70 (12%)	0.08
Body mass index	28.4 (5.4)	28.5 (5.9)	28.5 (5.1)	0.98
Current smoking	190 (20%)	61 (17%)	119 (21%)	0.14
Regular alcohol use	284 (29%)	87 (24%)	180 (32%)	0.02
Resting Systolic blood pressure (mmHg)	132.9 (21.1)	136.4 (22.5)	130.8 (20.2)	<0.001
Resting Diastolic blood pressure (mmHg)	74.6 (11.4)	74.3 (12.1)	74.9 (11.0)	0.44
NYHA Class III or IV	217 (22%)	99 (27%)	108 (19%)	0.003
**Medication use**				
Aspirin	705 (72%)	255 (70%)	421 (74%)	0.17
Statin	627 (64%)	230 (63%)	370 (65%)	0.54
ACE-inhibitor / ARB	499 (51%)	220 (60%)	262 (46%)	<0.001
Beta-blockers	565 (58%)	238 (65%)	307 (54%)	<0.001
Diuretics	288(30%)	152(42%)	125(22%)	<0.001
**Laboratory data**				
Cholesterol, mean(SD) (mg/dl)				
Total	177.5 (42.5)	176.1 (43.5)	178.3 (42.0)	0.44
HDL	45.6 (14.1)	44.4 (13.8)	46.2 (14.2)	0.06
LDL	104.3 (34.0)	103.3 (35.9)	105.1 (32.9)	0.44
Triglycerides	140.0 (125.1)	146.3 (119.5)	137.1 (129.4)	0.27
GFR, mean(SD) (ml/min/1.73m^2^)	70.6 (22.1)	51.9 (20.2)	81.7 (14.1)	<0.001
Albuminuria, mean(SD) (mg/g)	63.6 (278.0)	157.1 (440.8)	8.3 (5.6)	<0.001
Hemoglobin, mean(SD) (g/dL)	13.9 (1.4)	13.4 (1.5)	14.1 (1.3)	<0.001
**Cardiac function**				
LVEF (%)	61.7 (9.7)	59.9 (10.3)	62.7 (9.3)	<0.001
Diastolic function				
Normal	530 (54%)	153 (42%)	353 (62%)	<0.001
Impaired relaxation	227 (23%)	109 (30%)	109 (19%)	
Pseudonormal	60 (6%)	33 (9%)	27 (5%)	
Restrictive	45 (5%)	24 (7%)	18 (3%)	

*P-values for differences between CKD and non-CKD participants were determined by chi-squared for categorical variables, and Student’s t-test (age, lipid tests), T-test using Welch’s correction for unequal variances (BMI, systolic and diastolic blood pressure, eGFR, hemoglobin, LVEF) or Mann-Whitney tests (albuminuria) for continuous variables. Continuous data are summarized as mean (SD) or number participants (percent cohort). NYHA: New York Heart Association. ACE: angiotensin converting enzyme ARB: angiotensin receptor blocker. HDL high density lipoprotein. LDL low density lipoprotein. GFR glomerular filtration rate. LVEF Left ventricular ejection fraction

### Results of random forest regression analyses

**Fig 2:**
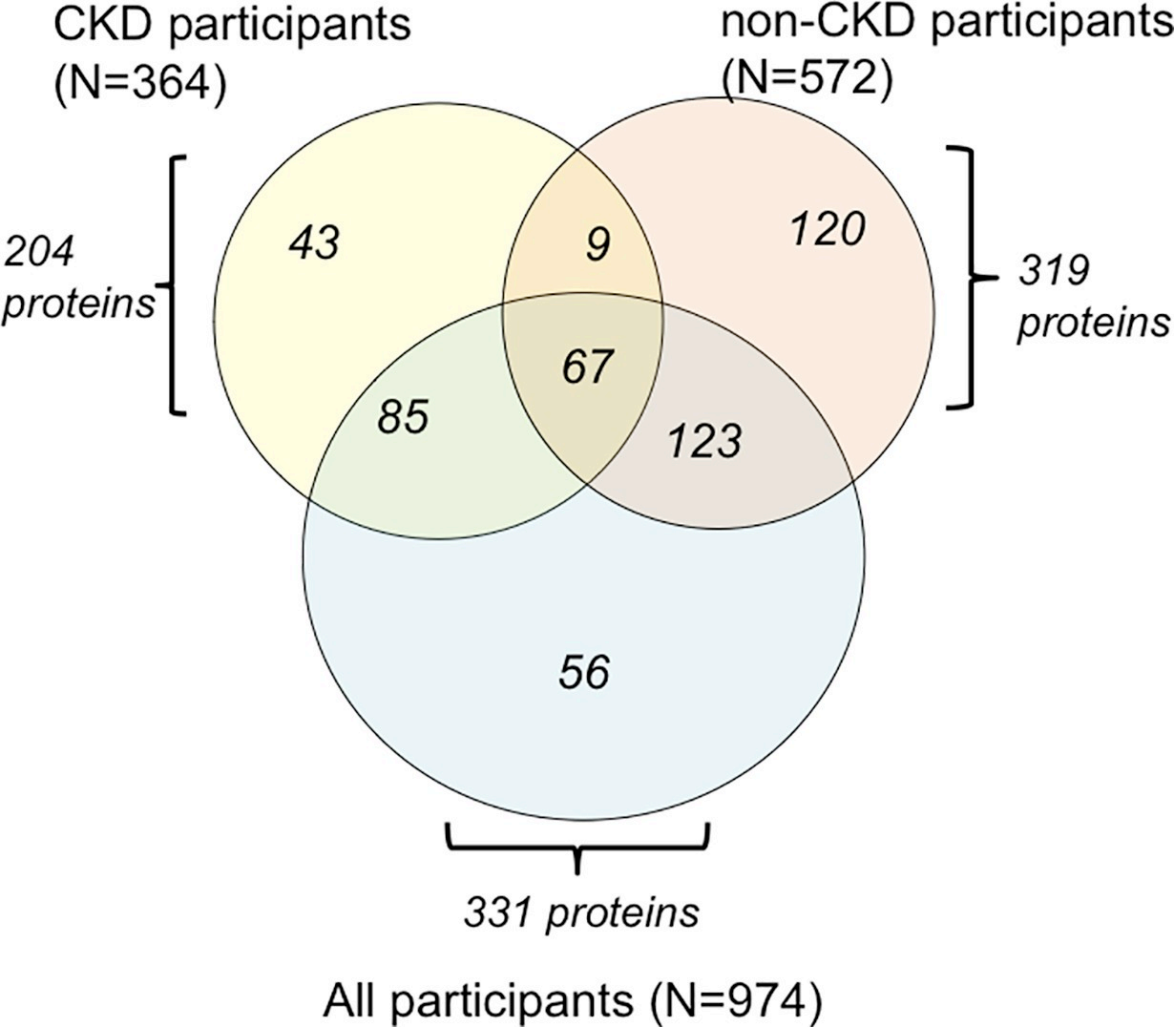
Results of random forest regression performed on full sample and sub-groups stratified by chronic kidney disease (See Attached File). Random survival forest regression was applied first to the full sample (N = 974 participants), then to the sub-groups with baseline CKD (N = 364 participants) and without baseline CKD (N = 572 participants). Forest regression of the full sample of participants yielded 331 proteins associated with HF outcomes (blue circle). Analysis of the sub-group with CKD resulted in 204 proteins (yellow circle), and analysis of the sub-group without CKD yielded 319 proteins (orange circle).

Among 974 participants included in these analyses, there were 192 heart failure hospitalizations over a mean(SD) follow-up of 7.0(3.2) years. Participants with CKD (N = 364) had a higher age-adjusted rate of heart failure hospitalization than those without CKD (N = 572) (66.17 cases per 1,000 person-years vs. 12.86 cases per 1,000 person-years, respectively). Random survival forest analysis of the full sample, CKD participant sub-group, and non-CKD participant sub-group yielded 331, 204 and 319 proteins associated with HF, respectively. As shown in **Fig 2 (below)**, prognostic proteins resulting from these three separate forest regressions only partially overlapped. There were 43 proteins that emerged as prognostic factors among the CKD subgroup of participants, but were not selected by forest regression of the full sample or the non-CKD sub-group. These 43 proteins and the full list of 204 proteins resulting from forest regression in the CKD sub-group are listed in **S2 Table.** As a sensitivity analysis, we performed Cox LASSO on the set of proteins selected in the random forest for the full sample as potential predictors of heart failure hospitalization. We found the same ten proteins in the Cox LASSO models when applied to the full cohort or the CKD-subgroup—in the full cohort, we also found one additional protein. The consistent findings between the whole group and CKD subgroup support that variable selection and fine tuning of lambda value were correctly performed.

### Interaction with CKD

For each of the 331 proteins generated from the random forest regression of the full sample of 974 participants, we calculated the Cox survival hazard ratio, adjusted for age and eGFR, and then tested these associations for an interaction with CKD. CKD had a significant interaction with 43 proteins at a significance of p<0.05. There were two CKD interactions with Bonferroni-corrected statistical significance: Spondin-1 (CKD HR, [95%CI] 1.11[1.04, 1.19], non-CKD HR, [95%CI] 2.16 [1.63, 2.86], p-value for interaction 1.4 x10^-6^; and Inhibin beta A chain (CKD HR, [95%CI] 0.82 [0.68, 0.99], non-CKD HR, [95%CI] 1.40 [1.17, 1.68]], p-value for interaction 8.6 x10^-5^. NOTCH1 was significantly associated with lower risk of heart failure in the CKD sub-group, but it was not significantly associated with HF outcomes in the non-CKD sub-group (CKD HR, [95%CI] 0.67 [0.56, 0.81], non CKD HR, [95%CI] 1.07 [0.82, 1.39], p-value for interaction 0.007. CD109 antigen showed a similar pattern of lower risk among participants with CKD, although CD109 was not selected by forest regression, and its hazard ratio among CKD participants was not significant. Vascular endothelial growth factor receptor 3, although it was not selected by random forest regression, was associated with higher risk of heart failure among patients with CKD (HR 1.4 [95%CI 1.16, 1.69]), and further, this was significantly higher risk than among patients without CKD (HR 1.04 [0.79, 1.37]) (p-value for CKD interaction 0.049). **(S3 Table)** Among patients in the CKD sub-group, we also performed an interaction analysis with DM for each of the 10 proteins selected by LASSO, and found no significant interactions (p>0.1 for all 10 proteins).

### Cox LASSO regression and multivariable analysis

Using the set of 204 proteins generated from random forest analyses of the CKD sub-group, we performed Cox LASSO regression to select proteins with the strongest associations with HF hospitalizations. Ten proteins remained in the final Cox LASSO model; eight were associated with higher risk of HF, and two were associated with lower risk of HF. Cox proportional hazards analyses (without LASSO) were then conducted separately for each of these 10 proteins. In unadjusted Cox models, four proteins qualified as statistically significant by a Bonferroni-corrected p-value < 0.05/204 (2.5x10^-4^). Angiopoietin-2 (ANGPT2) was associated with higher risk of HF(HR[95%CI] 1.45[1.33, 1.59]), as was Spondin-1 (HR[95%CI] 1.45[1.33, 1.59]), while tartrate-resistant acid phosphatase type 5 (TRAP5) (HR[95%CI] 0.65[0.53, 0.78]) and neurogenis locus notch homolog protein 1 (NOTCH1) (HR[95%CI] 0.67[0.55, 0.80]) were associated with lower risk. ANGPT2 and TRAP5 associations retained Bonferroni-level significance after adjustment for age, eGFR and baseline HF. The prognostic associations of NOTCH1 and Spondin-1 were attenuated after adjustment for history of heart failure, but remained significant at p<0.01. **(Table 2)** Further adjustment for diabetes and hypertension did not substantially attenuate hazard ratios for ANGPT2, Spondin-1, TRAP5 or NOTCH1.

**Table 2:** Hazard ratios and 95% confidence interval per 1-SD predicting time to heart failure hospitalization among those with chronic kidney disease* (N = 364), heart and soul study.

	Model 1	p-value	Model 2	p-value	Model 3	p-value	Model 4	p-value
**Higher heart failure risk**								
Angiopoietin-2	1.45 (1.33, 1.59)	3.33E-16	1.48 (1.35, 1.63)	0.0238	1.45 (1.31, 1.60)	0.0446	1.41 (1.28, 1.55)	7.08E-12
C-C motif chemokine 18	1.25 (1.11, 1.42)	0.000411	1.25 (1.10, 1.42)	0.00057	1.21 (1.05, 1.38)	0.00654	1.19 (1.04, 1.37)	0.01287
C-X-C motif chemokine 13	1.17 (1.07, 1.28)	0.000513	1.17 (1.07, 1.29)	0.00051	1.21 (1.10, 1.33)	0.000146	1.18 (1.07, 1.29)	0.000669
Spondin-1	1.13 (1.06, 1.20)	0.000201	1.11 (1.04, 1.19)	0.00137	1.11 (1.04, 1.19)	0.00295	1.11 (1.04, 1.19)	0.002096
TATA-box-binding protein	1.11 (1.02, 1.20)	0.0126	1.10 (1.01, 1.19)	0.0249	1.10 (1.02, 1.19)	0.017	1.11 (1.02, 1.20)	0.011858
Kallikrein-7	1.11 (1.03, 1.18)	0.00331	1.11 (1.04, 1.19)	0.00219	1.10 (1.02, 1.17)	0.00851	1.08 (1.01, 1.15)	0.027749
Hepatocyte growth factor	1.09 (1.02, 1.17)	0.0121	1.10 (1.03, 1.18)	0.00561	1.11 (1.04, 1.19)	0.00287	1.13 (1.05, 1.21)	0.000865
Carbohydrate sulfotransferase 2	1.05 (0.99, 1.11)	0.119	1.05 (0.99, 1.12)	0.1097	1.09 (1.02, 1.16)	0.014728	1.07 (1.00, 1.14)	0.03713
**Lower heart failure risk**								
Tartrate-resistant acid phosphatase type 5	0.65 (0.53, 0.78)	7.00E-06	0.65 (0.54, 0.79)	1.58E-05	0.66 (0.55, 0.80)	1.91E-05	0.68 (0.56, 0.82)	4.30E-05
Neurogenis locus notch homolog protein 1	0.67 (0.55, 0.80)	1.69E-05	0.66 (0.55, 0.79)	9.23E-06	0.67 (0.56, 0.81)	4.01E-05	0.72 (0.60, 0.88)	0.00106

Model 1—crude model; Model 2—adjusted for age (years); Model 3—adjusted for age (years) and eGFR; Model 4—adjusted for age, eGFR, and history of heart failure.

*Chronic kidney disease defined as eGFR<60 or albuminuria > 30 mg/g.

### Correlations of markers with eGFR and albuminuria

We examined whether the ten proteins selected after Cox LASSO regression in the CKD sub-group were correlated with renal function in the full sample (N = 974 participants). Five of the proteins associated with higher risk correlated with worse renal function (either lower eGFR or higher albuminuria): angiopoietin-2 (ANGPT2), C-C motif chemokine 18 (CCL18), C-C motif chemokine 13 (CCL13), Spondin-1(SPON1) and hepatocyte growth factor. Conversely, the two proteins associated with lower risk were associated with better renal function: Tartrate-resistant acid phosphatase type 5 (TRAP5) and Neurogenis locus notch homolog protein 1 (NOTCH1) **(Table 3).**

**Table 3:** Pearson's correlation coefficients between proteins predicting heart failure and egfr, or albuminuria in full sample*.

	Molecular Weight (kDa)	eGFR (ml/min/1.73m^2^)	Albuminuria (mg/g Cr)
**Higher heart failure risk**			
Angiopoietin-2	57	**-0.28 (<0.001)**	**0.12 (<0.001)**
C-C motif chemokine 18	7.8	**-0.36 (<0.001)**	**0.09 (0.006)**
C-X-C motif chemokine 13	13	**-0.10 (0.001)**	0.02 (0.47)
Spondin-1	27	**-0.21 (<0.001)**	**0.07 (0.049)**
TATA-box-binding protein	38	-0.02 (0.51)	-0.01 (0.75)
Kallikrein-7	86	-0.06 (0.06)	-0.004 (0.89)
Hepatocyte growth factor	103	-0.01 (0.81)	**0.08 (0.01)**
Carbohydrate sulfotransferase 2	53	0.04 (0.22)	-0.01 (0.79)
**Lower heart failure risk**			
Tartrate-resistant acid phosphatase type 5	35	**0.17 (<0.001)**	0.05 (0.14)
Neurogenis locus notch homolog protein 1	120	**0.24 (<0.001)**	**-0.08 (0.02)**

*Proteins were selected in survival random forests and Cox LASSO regression models predicting heart failure hospitalization in those with CKD. Pearson's correlation coefficients were estimated between these proteins and eGFR or Albuminuria in the full study sample. LASSO: Least absolute shrinkage and selection operator. eGFR: estimated glomerular filtration rate.

### Pathway analyses

**Fig 3:**
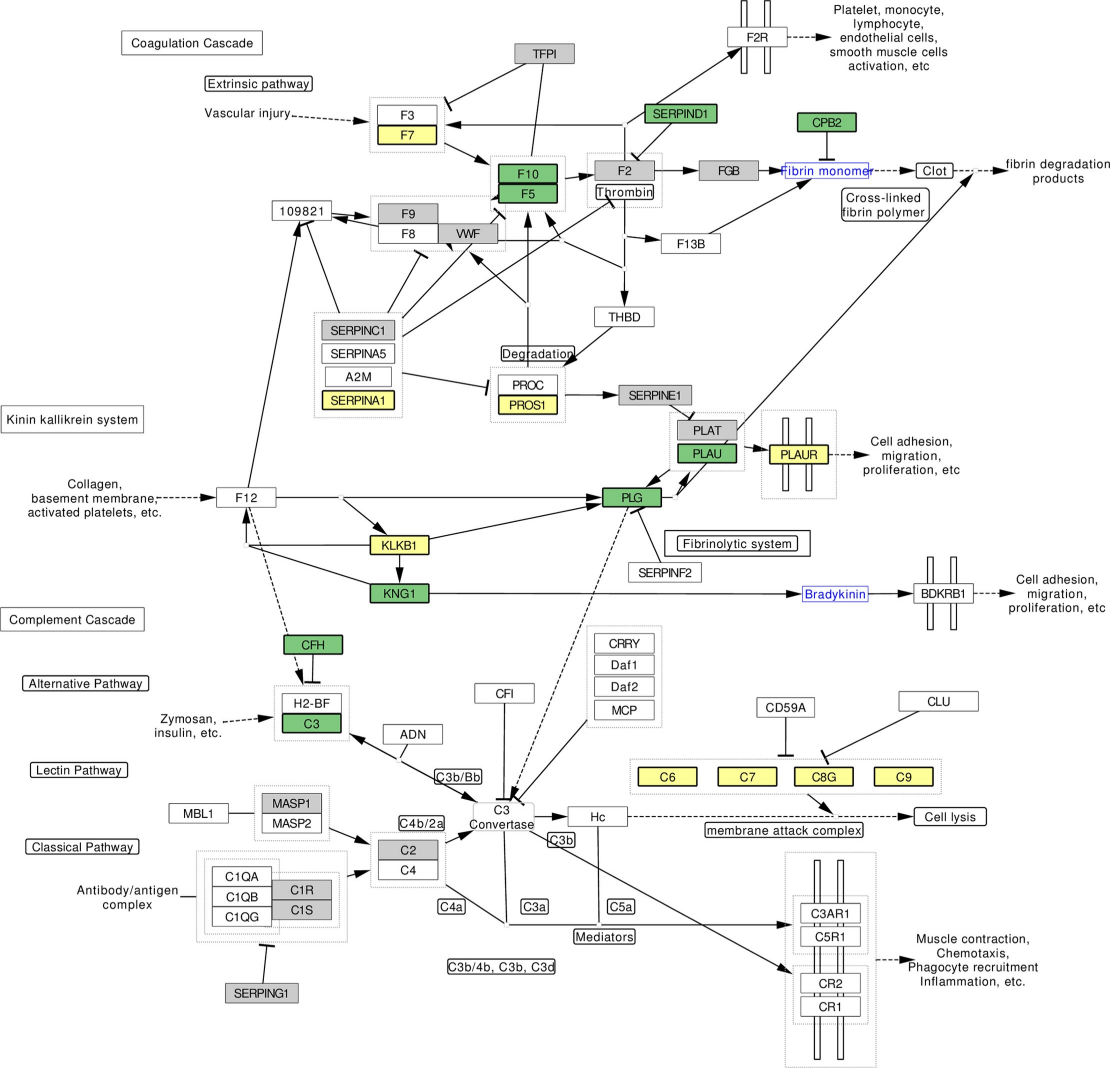
Mapping Clinical Outcome Information to the Complement and Coagulation Pathway (See Attached File). The Coagulation and Complement pathway is shown in this diagram, (source: https://www.wikipathways.org/index.php/Pathway:WP558.) Colored boxes represent results from random forest survival analyses. Gray boxes represent proteins associated with heart failure when the full sample was analyzed. Green boxes represent proteins that were associated with HF after analysis of the non-CKD sub-group. Yellow boxes represent proteins associated with HF after analysis of the CKD sub-group, including F7(Coagulation factor VII precursor), SERPINA1(Alpha-1-antitrypsin precursor), PROS1(Vitamin K dependent protein S precursor), PLAUR(Urokinase plasminogen activator surface receptor), KLKB1(Plasma kallikrein precursor), Complement(C)6, C7, C8-gamma chain, C9. Coagulation factors 3, 8, 12 and MASP2 were not measured.

We performed functional enrichment on subsets of proteins derived from forest analysis of proteins in three participant samples: a) the full sample, b) participants with CKD and c) participants without CKD. In both of these participant sub-groups we performed functional enrichment of the proteins associated with HF using two systems: Gene Ontology (geneontology.org) and Wikipathways (wikipathways.org). All 1068 proteins were identified in each of these two systems. Overall, proportional representation of pathways in the subset of 331 proteins associated with HF in the full sample was similar to the background of 1068 proteins. The most common Gene Ontology pathways in these two subsets, listed by pathway, (% of 331 proteins/% of 1068 proteins) were as follows: extracellular space (39%/34%), inflammatory response (10%/12%), regulation of response to wounding (11%/12%), cell surface (12%/11%), and coagulation (12%/15%). We then performed functional enrichment of the set of 204 proteins derived from random forest regression among persons in the CKD sub-group and compared this with the background of 1068 proteins. Using pathways defined by Wikipathways, the protein set derived from the CKD sub-group had a higher proportion of proteins in the Cardiac Hypertrophy (chi^2^ p = 0.17) and Complement/Coagulation pathway (chi^2^ p = 0.13), although these differences did not reach statistical significance. We mapped proteins associated with HF among CKD participants to the Complement/Coagulation pathway. These proteins included Coagulation factor VII (F7), plasma kallikrein (KLKB1), alpha-1-antitrypsin (SERPINA1), Vitamin K-dependent protein S (PROS1), urokinase plasminogen activator surface receptor (PLAUR) and Complements 6–9. (**Fig 3, below)**

## Discussion

In this study, we utilized the SOMAscan aptamer-based proteomic assay to perform an agnostic search for novel proteins associated with HF hospitalizations in the Heart and Soul Study and to characterize protein-HF pathways and risk associations according to CKD status. Our analysis highlights 10 proteins associated with risk of HF in participants of Heart and Soul with baseline CKD, four of which are associated with HF at Bonferroni-level significance (p<2.5x10^-4^): ANGPT2, Spondin-1, TRAP5, and NOTCH1. These 4 proteins relate to angiogenesis or atherogenesis, and their associations persist at p<0.01 after adjustment for age, eGFR and history of HF. CKD is a significant interaction term in the NOTCH1-HF association, and this association may be particularly important for patients with CKD.

Patients with CKD have high rates of HF,[31] but we lack a mechanistic explanation for this phenomenon. Our goal was to identify novel proteins associated with the specific outcome of HF hospitalizations, and to examine whether any of these associations differed among sub-groups with and without CKD. Previously, Ganz et al. developed a proteomics risk score for composite cardiovascular events (myocardial infarction, stroke, heart failure) and overall mortality (CVD9) in Heart and Soul and validated the risk score in the HUNT3 cohort.[22] Our analysis differs in its focus on HF hospitalizations, and our mechanistic interest in the role of CKD in protein-HF associations. It is notable that, despite these different analytic approaches, two proteins included in the 9 protein risk model developed by Ganz et al. were also selected to our final list of 10 proteins associated with heart failure among the CKD sub-group: Angiopoietin-2 and C-C motif chemokine 18. Three other proteins in the CVD9 model have roles in the complement/coagulation pathway, which showed a trend for higher representation among proteins selected in the CKD sub-group: complement 7, SERPINA3, SERPINF2. Thus, we feel our results both corroborate and expand on the prior analysis.

The NOTCH1 protein was associated with lower risk of HF in the CKD sub-group after multivariable adjustment for age, eGFR and HF. In the full sample, baseline CKD status had a significant interaction with the NOTCH1-HF association, suggesting that this marker may have particular importance for patients with CKD. The NOTCH1 pathway has central roles in endothelial cell senescence and atherogenesis.[32, 33] In angiogenesis, NOTCH1 in endothelial tip cells regulates angiogenic sprouting.[34, 35] Activation of NOTCH1 ameliorates cardiac injury after MI.[36] It is important to note that NOTCH1 is a cell membrane receptor, and we have measured it in the extracellular space. Further studies are needed to delineate whether serum levels correlate with NOTCH1 pathway activity.

We found TRAP5 to be associated with lower risk of HF hospitalization among participants with CKD after adjustment for age, eGFR and baseline HF. TRAP5 is a metalloenzyme found in osteoclasts and macrophages, known to be resistant to inhibition by tartrate and activated in acidic conditions.[37] It is commonly used as a histological marker of osteoclasts, but also has important functions in bone metabolism, immunity and cell growth and differentiation.[38] Inactivating mutations of the TRAP5 gene deregulate osteopontin signaling,[39] an important causal pathway of atherogenesis.[40, 41] It is interesting to note that CD109, a protein found in osteoclast precursors,[42] as well as activated platelets and T-cells,[43] was associated with lower risk of heart failure among CKD participants than among non-CKD participants, (p-value for CKD interaction = 0.049). CD109 did not appear in the list of proteins associated with heart failure generated by random forest regression.

Spondin-1 (SPON1), also known as F-spondin, is a secreted protein of the thrombospondin family, found primarily in the extracellular matrix. SPON1 deficient mice have upregulated bone deposition and higher bone mass.[44] High concentrations of F-spondin are found in the human ovary, where it is known to stimulate vascular smooth muscle cell proliferation.[45] Given the negative Spearman correlation of Spondin-1 with eGFR and albuminuria, it follows that Spondin-1 levels were lower in those with higher eGFR, yet the Spondin-1-HF association was significantly stronger in these non-CKD patients. This could be due to a saturation point of Spondin-1 or its effectors, and warrants further study.

ANGPT2 was associated with a higher risk of HF among patients with CKD, even after adjustment for age, eGFR and baseline HF. ANGPT2 inhibits angiogenesis when VEGF is low (as in CKD),[46, 47] but promotes angiogenesis when VEGF levels are high.[48] ANGPT2 is produced and stored in endothelial cells and is released when exposed to TNF, VEGF, or hypoxia.[49] Serum levels of ANGPT2 inversely correlate with eGFR and are elevated in patients with advanced stages of CKD.[50] Circulating ANGPT2 is associated with mortality among patients with heart failure with reduced ejection fraction[51] and is associated with early cardiovascular disease in children with end-stage renal disease on dialysis.[52] ANGPT2 is included in the proteomic risk score for composite cardiovascular events and death that was developed in Heart and Soul by Ganz et al.[22] Although it was not selected by random forest regression, vascular endothelial growth factor receptor 3 is another angiogenic factor associated with higher risk of heart failure among patients with CKD, and this association was marginally stronger in those with CKD than in those without CKD (p-value for CKD interaction = 0.049). These findings support further work to explore the impact of angiogenic factors on heart failure among patients with CKD.

Overall, among the 10 proteins selected in the CKD sub-group, serum levels of 6 proteins correlated with eGFR. Of these 6, those associated with higher risk of HF correlated with lower eGFR, and those associated with lower risk of HF correlated negatively with higher eGFR. One challenge in researching biomarkers of cardiovascular disease in patients with CKD is that serum levels of smaller molecules (typically <30kDa) are influenced directly by glomerular filtration, and it can be difficult to determine whether a smaller molecule is simply a marker of CKD or if it plays an active biological role in causing disease. Direct glomerular filtration could influence the levels of three of the smaller proteins found to have negative correlations with eGFR (C-C motif chemokine 18, C-X-C motif chemokine 13, and Spondin-1). The positive correlations of TRAP5 and NOTCH1 with eGFR would not be explained by this mechanism. We show that adjustment for eGFR had minimal effect on HF associations of the 10 selected proteins. In particular, the associations of TRAP5 and NOTCH1 with lower risk of HF remained significant at Bonferroni-level p-value after adjustment for eGFR. These observations suggest that TRAP5 and NOTCH1 may have active biological roles in the development or recurrence of HF. Further analyses of these markers in CKD and non-CKD HF cohorts are needed to confirm these hypotheses.

Functional enrichment illustrates the relative importance of biological pathways and ontology terms, and in the case of pathways, illustrates the upstream or downstream position of a particular protein in the pathway. By comparing functional enrichment of the 204 proteins selected from random survival forest regression among patients with CKD to the background set of 1068 proteins included in our analysis, we found that Cardiac Hypertrophy and Complement/Coagulation pathways were somewhat more prominent (although differences did not reach statistical significance), relative to the background of 1068 proteins. These results are consistent with what we know about cardiovascular disease in patients with CKD, in whom left ventricular hypertrophy[53] and abnormal bleeding and coagulation[54, 55] are common. However, it is important to consider that stable coronary artery disease was an inclusion criteria for the Heart and Soul Study. In this specialized population, recurrent myocardial ischemia related to atherosclerotic disease may be closely related to recurrent or incident heart failure events, and this may account for our observation of a trend for higher representation of proteins in the complement and coagulation pathway. It is also notable that the aptamer platform used for this study did not measure all components of the complement / coagulation cascade; further analysis of these pathways should be performed with aptamer platforms that cover a larger range of proteins. We have provided a novel perspective on pathway analysis by incorporating clinical outcome information with the map of the coagulation pathway, to illustrate an approach that may help us to understand differences in mechanisms of heart failure between subgroups of patients with and without CKD.

There are several important limitations to the current analysis. Patients in the Heart and Soul Study all had coronary artery disease, and thus our results may not generalize to HF of non-ischemic origin. Further studies would be needed to understand if the proteins selected in our analysis are markers of non-ischemic heart failure. Another important step in future research will be to examine how proteomics differ between patients with heart failure preserved ejection fraction and heart failure reduced ejection fraction, which we did not differentiate. Future studies might also clarify whether proteomic risk factors differ in patients on different preventive treatments, such as statins. Etiology of CKD was not adjudicated in Heart and Soul, and it is possible that mechanisms of cardiovascular disease differ among CKD patients with different etiologies of kidney disease—e.g., hypertension, or glomerulonephritis. Further studies in cohorts with a large, more diverse sample of CKD patients might elucidate these differences. Conclusions from this study are limited prior to validation in larger cohorts. Our analysis included 1068 proteins, a small portion of the human proteome; there are likely to be other proteins essential to HF risk that were not measured. Functional enrichment performed on these HF risk factors relative to the background of the SOMAscan assay may also obscure pathways that might emerge if we could compare our subset of HF factors to a larger, random sample of the proteome. Further, since our proteomic investigation was limited to plasma, pathway analyses may not represent tissue-specific biological mechanisms. The core proteomic dataset we utilized was the same as for the prior analysis by Ganz et al.,[22] but we had slightly different criteria for excluding proteins from the analysis: we chose not to exclude proteins based on quality metrics, but we did exclude 28 proteins (including troponin I) not designated as ‘human’ targets in the Heart and Soul dataset. None of the 10 proteins selected by forest and Cox LASSO regression in the CKD sub-group were noted to have poor quality metrics in the prior analyses.

## Conclusions

Despite these limitations, our analysis revealed four proteins associated with HF risk among patients with CKD in Heart and Soul. In particular, NOTCH1, a regulator of angiogenesis, was associated with lower HF risk among patients with CKD, but not among patients without CKD. We show that integrating CKD phenotype information, survival analyses and network analysis can highlight clinically relevant defects in specific biological pathways and might focus further investigations on developing therapeutic targets in patients with CKD. Validation in additional cohorts with adequate numbers of patients with CKD is needed to confirm associations of novel proteins with HF.

## Supporting information

S1 File Expanded methods. (DOCX)

S1 Table Proteins excluded from current analysis. We excluded 28 proteins from the current analysis that were labeled in the Heart and Soul proteomic dataset as being assayed with non-human aptamers.
(DOCX)

S2 Table Proteins associated with heart failure selected by random survival forest regression among ckd participants of heart and soul. (DOCX)

S3 Table Protein—Heart failure associations with interactions by CKD status. (DOCX)
