# Supplementary material for: Proteomic analysis of heart failure hospitalization among patients with chronic kidney disease: The Heart and Soul Study
Source: PLoS One. 2018 Dec 17;13(12):e0208042. doi: 10.1371/journal.pone.0208042 (PMC6296511; doi:10.1371/journal.pone.0208042)
Supplement: S1 File — (DOCX) [file pone.0208042.s001.docx]

**Expanded Methods**

Heart and Soul Study

*Study population*

The Heart and Soul study included 1024 participants with stable coronary artery disease recruited between 2000 and 2002 from two Department of Veterans Affairs Medical Centers, 1 university medical center, and 9 public health clinics in San Francisco between 2000 and 2002. To be included, participants had to have stable coronary artery disease, defined as clinical history of myocardial infarction, clinical history of revascularization, >50% angiographic stenosis, or inducible ischemia during treadmill or pharmacological stress. History of MI in the previous 6 months, inability to walk 1 block, or plans to move out of the local area within 3 years were exclusion criteria. All participants provided written informed consent to participate in the Heart and Soul Study. In these analyses, we included 974 participants with assessment of proteomics at baseline. Veterans Affairs Institutional Review Boards at each site approved the study protocol. All records were anonymized before statistical analysis.

*Covariates*

Study participants completed questionnaires to report age, gender, race, tobacco or alcohol use, history of heart failure, myocardial infarction, as well as non-cardiac medical history. Assays for cholesterol were performed on fasting baseline plasma samples, along with additional baseline assays as previously described.^1-3^Cystatin C was measured with a BNII nephelometer (Dade Behring, Inc., Deerfield, Ill., USA) using a particle-enhanced immunonephelometric assay (N Latex Cystatin C; Dade Behring, Inc.). eGFR was calculated from cystatin C and creatinine, using the Chronic Kidney Disease Epidemiology Collaboration equations.^4, 5^ Urinary albumin was measured by nephelometer, and urine creatinine by the rate Jaffe method. Two-dimensional transthoracic echocardiography was performed on each participant at rest, using the Acuson Sequoia ultrasound system (Siemens Medical Solutions USA, Inc., Mountain View, CA, USA), and read by one expert cardiologist who was blinded to all clinical data.^6^ LV mass index was estimated using the truncated ellipsoid method for mass, divided by body surface area. ^7^ Left ventricular ejection fraction was calculated by the biplane method of disks. Diastolic function was classified as normal, impaired, pseudonormal or restrictive filling on mitral flow velocity.^8^

*Adjudication of heart failure*

Adjudication of CVD outcomes in H&S is previously described.^3^ In brief, annual telephone interviews were conducted with participants or their proxies regarding recent emergency room visits, hospitalizations, or death. Two independent and blinded adjudicators reviewed events, joined by a third reviewer if they did not agree.^9^ HF hospitalization had to incur at least 1-night overnight admission for a syndrome with at least 2 of the major Framingham criteria for heart failure ^10^, as well as signs of decreased cardiac output manifested as hypoperfusion (in the absence of dehydration or sepsis), or peripheral or pulmonary edema requiring diuretic therapy.^2^

Complete Statistical Methods

The study sample consisted of 974 Heart and Soul Study participants who had proteomics measured at baseline; 936 of these participants had measures of eGFR and ACR at baseline. We selected 1068 aptamers that were designated “human” and “proteins” and evaluated these as potential predictors in the random survival forests. Each protein was transformed to a standard normal distribution by centering the protein around its mean and dividing by its standard deviation. After normal standardization of the 1068 proteins, random survival forests^11^ were used to model time to HF hospitalization. We compared prediction error rates using Harrell’s concordance index^12^ to determine optimal settings of parameters for the lowest prediction error rate in the forest model. The final ensemble consisted of 1000 trees; in each tree, the number of candidate variables at each split was set to the square root of the total number of proteins (n=33), and the node size was set to a minimum of three HF hospitalization events in a terminal node. For each protein, we calculated the minimal depth statistic. This parameter is a comparative measure of the predictive quality of each protein relative to the root node, representing the first split of the tree. Smaller values of minimal depth indicate better predictive qualities. The average minimal depth was calculated from all proteins, and proteins with minimal depth values below the average were selected as the top predictive variables. Forest regression analyses were repeated in sub-groups of patients with CKD (N=364) and without CKD (N=572), selecting the top predictive proteins for CKD and non-CKD, separately.

We then applied two methods to prioritize and categorize the proteins chosen by forest regression in the CKD sub-group: LASSO (least absolute shrinkage and selection operator) regression and pathway analyses. The candidate proteins selected in the CKD-subgroup were further reduced by fitting a Cox LASSO regression model predicting heart failure in those with CKD. To find the optimal value of the shrinkage parameter (lambda) for the Cox LASSO model, we averaged the values for lambda obtained from 100 replications of 10-fold cross validation, choosing the lambda with the smallest mean cross-validated error from each replication. As a sensitivity analysis, we repeated the Cox LASSO regression analyses in the CKD-subgroup instead using the set of proteins selected in the random forest for the full sample as potential predictors of heart failure hospitalization. Using this method on both subsets of proteins selected in the full sample and the CKD-subgroup, we found the same ten proteins in the Cox LASSO models when applied to the full cohort or the CKD-subgroup. In the full cohort, we also found one additional protein. The consistent findings between the whole group and CKD subgroup support that variable selection and fine tuning of lambda value were correctly performed. After eliminating less predictive proteins by the Cox LASSO method, we fit separate Cox proportional hazards regression models for each of the final LASSO selected proteins predicting time to HF hospitalization in the CKD subgroup, successively adjusting for baseline measures of age (continuous, years), eGFR (continuous, ml/min/m^2^) and history of heart failure (yes / no). At this stage, the threshold of significance was the Bonferroni-corrected p-value 0.05/N=204 (N=number of proteins associated with HF among patients with CKD in random forest regression). Pearson’s correlation coefficients were estimated between proteins selected in the CKD subgroup and eGFR or albuminuria in the full sample of participants. Additionally, we tested interactions between the top predictive proteins selected in random survival forest model for the full sample and CKD (yes / no), with significance threshold for the interaction term at p<0.05. Hazard ratios for the relative hazard of heart failure were estimated stratified by CKD status for the statistically significant interactions. An organizational chart for these analyses is shown in **Figure 1.** Data management and statistical analyses were conducted using R version 3.3.0.^13^

While the methods integrated into our analysis have complementary strengths, each does also have limitations. Random forest regression models have several advantages, including the excellent predictive qualities of the model, the availability of the minimal depth statistic that would select a subset of proteins for the LASSO models,^14, 15^ and the capacity to incorporate interactions between variables and non-linear relationships in the model.^16^ Additionally, this initial selection step generated lists of proteins amenable to functional enrichment in an unbiased manner. We chose Cox LASSO models to select a smaller set of proteins because they are less susceptible to biases inherent in stepwise methods.^17^ However, the Cox LASSO model captures linear relationships, and we may be overlooking potential interactions between proteins that predict heart failure among participants with CKD. Another limitation of LASSO models is the potential to select different sets of variables based on the value of the shrinkage parameter, lambda. To stabilize the value of lambda, we performed several replications of cross-validation and took the average for the values of lambda from each replication.

*Pathway analyses*

Functional enrichment allows one to categorize proteins or genes into known biological pathways. Sets of proteins may be compared based on the proportion of proteins or genes that fall in given categories of biological function. For our analyses, we drew on information about the proteins’ functional category from Gene Ontology (geneontology.org) and WikiPathways (wikipathways.org) using Enrichr.^18^ We compared representation of functional categories between a) protein predictors of HF in the CKD group and b) the overall background of the SomaLogic assay proteins, using a chi^2^ significance threshold of p<0.2. Visualization of pathway analysis results allows us to map specific proteins of interest within a given pathway and locate the protein upstream or downstream of other prognostic proteins. We mapped the position of specific prognostic factors within pathways that were over-represented in the subset of proteins associated with HF among CKD participants using WikiPathways^19^ and Cytoscape.^20^

**References**

1. Ruo B, Rumsfeld JS, Hlatky MA, Liu H, Browner WS and Whooley MA. Depressive symptoms and health-related quality of life: the Heart and Soul Study. *Jama*. 2003;290:215-21.

2. Stevens SM, Farzaneh-Far R, Na B, Whooley MA and Schiller NB. Development of an echocardiographic risk-stratification index to predict heart failure in patients with stable coronary artery disease: the Heart and Soul study. *JACC Cardiovascular imaging*. 2009;2:11-20.

3. Whooley MA, de Jonge P, Vittinghoff E, Otte C, Moos R, Carney RM, Ali S, Dowray S, Na B, Feldman MD, Schiller NB and Browner WS. Depressive symptoms, health behaviors, and risk of cardiovascular events in patients with coronary heart disease. *Jama*. 2008;300:2379-88.

4. Inker LA, Schmid CH, Tighiouart H, Eckfeldt JH, Feldman HI, Greene T, Kusek JW, Manzi J, Van Lente F, Zhang YL, Coresh J, Levey AS and Investigators C-E. Estimating glomerular filtration rate from serum creatinine and cystatin C. *N Engl J Med*. 2012;367:20-9.

5. Levey AS, Stevens LA, Schmid CH, Zhang YL, Castro AF, 3rd, Feldman HI, Kusek JW, Eggers P, Van Lente F, Greene T, Coresh J and Ckd EPI. A new equation to estimate glomerular filtration rate. *Ann Intern Med*. 2009;150:604-12.

6. Ix JH, Shlipak MG, Chertow GM, Ali S, Schiller NB and Whooley MA. Cystatin C, left ventricular hypertrophy, and diastolic dysfunction: data from the Heart and Soul Study. *Journal of cardiac failure*. 2006;12:601-7.

7. Schiller NB, Shah PM, Crawford M, DeMaria A, Devereux R, Feigenbaum H, Gutgesell H, Reichek N, Sahn D, Schnittger I and et al. Recommendations for quantitation of the left ventricle by two-dimensional echocardiography. American Society of Echocardiography Committee on Standards, Subcommittee on Quantitation of Two-Dimensional Echocardiograms. *Journal of the American Society of Echocardiography : official publication of the American Society of Echocardiography*. 1989;2:358-67.

8. Wang EY, Dixson J, Schiller NB and Whooley MA. Causes and Predictors of Death in Patients With Coronary Heart Disease (from the Heart and Soul Study). *The American journal of cardiology*. 2017;119:27-34.

9. Schopfer DW, Regan M, Heidenreich PA and Whooley MA. Depressive Symptoms, Cardiac Disease Severity, and Functional Status in Patients With Coronary Artery Disease (from the Heart and Soul Study). *The American journal of cardiology*. 2016;118:1287-1292.

10. Ho KK, Pinsky JL, Kannel WB and Levy D. The epidemiology of heart failure: the Framingham Study. *J Am Coll Cardiol*. 1993;22:6A-13A.

11. Ishwaran H KU, Chen X, Minn AJ. Random Survival Forests for High-Dimensional Data. *Statistical Analysis and Data Mining*. 2010;4:115-132.

12. Harrell FE, Jr., Califf RM, Pryor DB, Lee KL and Rosati RA. Evaluating the yield of medical tests. *Jama*. 1982;247:2543-6.

13. Team RC. R: A Language and Environment for Statistical Computing. 2017.

14. Ishwaran H KU, Blackstone E, Lauer M. Random Survival Forests. *The Annals of Applied Statistics*. 2008;2:841-860.

15. Ishwaran H KU, Gorodeski E, Minn A, Lauer M. High-dimensional variable selection for survival data. . *Journal of the American Statistical Association*. 2010;105:205-217.

16. Lunetta KL, Hayward LB, Segal J and Van Eerdewegh P. Screening large-scale association study data: exploiting interactions using random forests. *BMC Genet*. 2004;5:32.

17. Tibshirani R. The lasso method for variable selection in the Cox model. *Statistics in medicine*. 1997;16:385-95.

18. Kuleshov MV, Jones MR, Rouillard AD, Fernandez NF, Duan Q, Wang Z, Koplev S, Jenkins SL, Jagodnik KM, Lachmann A, McDermott MG, Monteiro CD, Gundersen GW and Ma'ayan A. Enrichr: a comprehensive gene set enrichment analysis web server 2016 update. *Nucleic Acids Res*. 2016;44:W90-7.

19. Kutmon M, Riutta A, Nunes N, Hanspers K, Willighagen EL, Bohler A, Melius J, Waagmeester A, Sinha SR, Miller R, Coort SL, Cirillo E, Smeets B, Evelo CT and Pico AR. WikiPathways: capturing the full diversity of pathway knowledge. *Nucleic Acids Res*. 2016;44:D488-94.

20. Shannon P, Markiel A, Ozier O, Baliga NS, Wang JT, Ramage D, Amin N, Schwikowski B and Ideker T. Cytoscape: a software environment for integrated models of biomolecular interaction networks. *Genome Res*. 2003;13:2498-504.
