# Supplementary material for: Proteomic analysis of heart failure hospitalization among patients with chronic kidney disease: The Heart and Soul Study
Source: PLoS One. 2018 Dec 17;13(12):e0208042. doi: 10.1371/journal.pone.0208042 (PMC6296511; doi:10.1371/journal.pone.0208042)
Supplement: S1 Table — We excluded 28 proteins from the current analysis that were labeled in the Heart and Soul proteomic dataset as being assayed with non-human aptamers. (DOCX) [file pone.0208042.s002.docx]

**Supplemental Table 1. Proteins Excluded From Current Analysis**

We excluded 28 proteins from the current analysis that were labeled in the Heart and Soul proteomic dataset as being assayed with non-human aptamers.

| **Target** | **TargetFullName** | **UniProt** |
| --- | --- | --- |
| HPV E7 Type 16 | Protein E7_HPV16 | P03129 |
| HPV E7 Type18 | Protein E7_HPV18 | P06788 |
| HIV-2 Rev | Protein Rev_HV2BE | P18093 |
| KDGL_ECOLI | Diacylglycerol kinase_ECOLI | P0ABN1 |
| NEUA_NEIME | N-acylneuraminate cytidylyltransferase_NEIME | P0A0Z8 |
| GLRX1_ECOLI | Glutaredoxin-1_ECOLI | P68688 |
| MDH_THETH | Malic dehydrogenase_THETH | P10584 |
| KAD_GEOSE | Adenylate kinase_GEOSE | P27142 |
| YOPH_YEREN | Tyrosine-protein phosphatase YopH_YEREN | P15273 |
| HLA_STAAU | Alpha-hemolysin_STAAU | P09616 |
| GFP_AEQVI | Green fluorescent protein_AEQVI | P42212 |
| LUCI_PHOPY | Luciferin 4-monooxygenase_PHOPY | P08659 |
| HLA_STAAU | Alpha-hemolysin_STAAU | P09616 |
| NOH4_RHIML | Nodulation protein H_NOH4 | P06237 |
| EXE4_HELSU | Exendin-4_HELSU | P26349 |
| NIGB_SAMNI | Nigrin b_SAMNI | P33183 |
| APOA1_MOUSE | Apolipoprotein A-I_MOUSE | Q00623 |
| GFP_AEQVI | Green fluorescent protein_AEQVI | P42212 |
| MEL_VESMG | Melittin_VESMG | P68408 |
| C34 gp41 HIV Fragment | gp41 C34 peptide, HIV | Q70626 |
| GCKR_MACFA | Glucokinase Regulatory Protein_MACFA | None |
| PCSK9_MOUSE | Proprotein convertase subtilisin/kexin type 9 | Q80W65 |
| PPIF_MOUSE | Peptidyl-prolyl cis-trans isomerase F, mitochondrial_MOUSE | Q99KR7 |
| FABP | Fatty acid-binding protein, heart | P05413 |
| NGAL_RAT | Neutrophil gelatinase-associated lipocalin_RAT | P30152 |
| Troponin I, skeletal, fast twitch | Troponin I, fast skeletal muscle | P48788 |
| Troponin I | Troponin I, cardiac muscle | P19429 |
| ANP | Atrial natriuretic factor | P01160 |
