# Supplementary material for: Proteomic analysis of heart failure hospitalization among patients with chronic kidney disease: The Heart and Soul Study
Source: PLoS One. 2018 Dec 17;13(12):e0208042. doi: 10.1371/journal.pone.0208042 (PMC6296511; doi:10.1371/journal.pone.0208042)
Supplement: S2 Table — (DOCX) [file pone.0208042.s003.docx]

**Supplemental Table 2. Proteins Associated with Heart Failure Selected by Random Survival Forest Regression Among CKD Participants of Heart and Soul.**

Asterisk (**) labels proteins that emerged in the CKD sub-group but not the full sample or non CKD sub-group.

| **Target** | **TargetFullName** | **UniProt** |
| --- | --- | --- |
| C4b** | Complement C4b | P0C0L4 P0C0L5 |
| C9** | Complement component C9 | P02748 |
| C8 | Complement component C8 | P07357,P07358,P07360 |
| CXCL16, soluble | C-X-C motif chemokine 16 | Q9H2A7 |
| SAP | Serum amyloid P-component | P02743 |
| Factor I | Complement factor I | P05156 |
| Myeloperoxidase | Myeloperoxidase | P05164 |
| Angiopoietin-2 | Angiopoietin-2 | O15123 |
| GA733-1 protein | Tumor-associated calcium signal transducer 2 | P09758 |
| HSP 90a/b** | Heat shock protein HSP 90-alpha/beta | P07900 P08238 |
| IL-12 Rb1 | Interleukin-12 receptor subunit beta-1 | P42701 |
| PKC-A | Protein kinase C alpha type | P17252 |
| suPAR** | Urokinase plasminogen activator surface receptor | Q03405 |
| BCMA** | Tumor necrosis factor receptor superfamily member 17 | Q02223 |
| HGF | Hepatocyte growth factor | P14210 |
| HSP 60 | 60 kDa heat shock protein, mitochondrial | P10809 |
| MIA | Melanoma-derived growth regulatory protein | Q16674 |
| NPS-PLA2 | Phospholipase A2, membrane associated | P14555 |
| OSM** | Oncostatin-M | P13725 |
| Persephin | Persephin | O60542 |
| Protein S | Vitamin K-dependent protein S | P07225 |
| Sonic Hedgehog | Sonic hedgehog protein | Q15465 |
| Activin A | Inhibin beta A chain | P08476 |
| FGF-18 | Fibroblast growth factor 18 | O76093 |
| FGF-20 | Fibroblast growth factor 20 | Q9NP95 |
| Lactoferrin | Lactotransferrin | P02788 |
| LD78-beta** | C-C motif chemokine 3-like 1 | P16619 |
| MMP-7 | Matrilysin | P09237 |
| Apo B | Apolipoprotein B | P04114 |
| EDA | Ectodysplasin-A, secreted form | Q92838 |
| Kallikrein 11 | Kallikrein-11 | Q9UBX7 |
| Kallikrein 4** | Kallikrein-4 | Q9Y5K2 |
| Met | Hepatocyte growth factor receptor | P08581 |
| Ubiquitin+1 | Ubiquitin+1, truncated mutation for UbB | P62979 |
| CHK1 | Serine/threonine-protein kinase Chk1 | O14757 |
| Karyopherin-a2 | Importin subunit alpha-1 | P52292 |
| TBP | TATA-box-binding protein | P20226 |
| YES** | Tyrosine-protein kinase Yes | P07947 |
| a1-Antichymotrypsin** | Alpha-1-antichymotrypsin | P01011 |
| C7 | Complement component C7 | P10643 |
| Cardiotrophin-1 | Cardiotrophin-1 | Q16619 |
| Midkine | Midkine | P21741 |
| Cytochrome P450 3A4 | Cytochrome P450 3A4 | P08684 |
| MMP-8 | Neutrophil collagenase | P22894 |
| Protein C | Vitamin K-dependent protein C | P04070 |
| BMP-7 | Bone morphogenetic protein 7 | P18075 |
| CD36 ANTIGEN | Platelet glycoprotein 4 | P16671 |
| ENA-78 | C-X-C motif chemokine 5 | P42830 |
| IL-18 Rb | Interleukin-18 receptor accessory protein | O95256 |
| MBL | Mannose-binding protein C | P11226 |
| NKp30 | Natural cytotoxicity triggering receptor 3 | O14931 |
| TGF-b R III** | Transforming growth factor beta receptor type 3 | Q03167 |
| a2-Antiplasmin | Alpha-2-antiplasmin | P08697 |
| bFGF | Fibroblast growth factor 2 | P09038 |
| Galectin-2 | Galectin-2 | P05162 |
| GFAP** | Glial fibrillary acidic protein | P14136 |
| MIP-1a** | C-C motif chemokine 3 | P10147 |
| ON | SPARC | P09486 |
| PARC | C-C motif chemokine 18 | P55774 |
| BAFF | Tumor necrosis factor ligand superfamily member 13B | Q9Y275 |
| Cathepsin B | Cathepsin B | P07858 |
| FGF-5** | Fibroblast growth factor 5 | P12034 |
| IgM | Immunoglobulin M | P01871 |
| LBP | Lipopolysaccharide-binding protein | P18428 |
| IgG | Immunoglobulin G | P01857 |
| Siglec-3 | Myeloid cell surface antigen CD33 | P20138 |
| ATS13** | A disintegrin and metalloproteinase with thrombospondin motifs 13 | Q76LX8 |
| Coagulation Factor VII** | Coagulation Factor VII | P08709 |
| WFKN1** | WAP, kazal, immunoglobulin, kunitz and NTR domain-containing protein 1 | Q96NZ8 |
| Granulysin | Granulysin | P22749 |
| HPLN1 | Hyaluronan and proteoglycan link protein 1 | P10915 |
| IDE | Insulin-degrading enzyme | P14735 |
| ASAH2 | Neutral ceramidase | Q9NR71 |
| TrATPase | Tartrate-resistant acid phosphatase type 5 | P13686 |
| URB | Coiled-coil domain-containing protein 80 | Q76M96 |
| BGN | Biglycan | P21810 |
| CD48** | CD48 antigen | P09326 |
| FCG2A/B | Low affinity immunoglobulin gamma Fc region receptor II-a/b | P12318 P31994 |
| RGMB | RGM domain family member B | Q6NW40 |
| Carbonic anhydrase 6 | Carbonic anhydrase 6 | P23280 |
| Carbonic anhydrase VII** | Carbonic anhydrase 7 | P43166 |
| CRDL1 | Chordin-like protein 1 | Q9BU40 |
| Kallikrein 7 | Kallikrein-7 | P49862 |
| PLK-1 | Serine/threonine-protein kinase PLK1 | P53350 |
| BFL1 | Bcl-2-related protein A1 | Q16548 |
| BSP | Bone sialoprotein 2 | P21815 |
| BTK** | Tyrosine-protein kinase BTK | Q06187 |
| CDK1/cyclin B** | Cyclin-dependent kinase 1:G2/mitotic-specific cyclin-B1 complex | P06493 P14635 |
| CSK21 | Casein kinase II subunit alpha | P68400 |
| HIPK3 | Homeodomain-interacting protein kinase 3 | Q9H422 |
| IL-18 Ra | Interleukin-18 receptor 1 | Q13478 |
| STK16 | Serine/threonine-protein kinase 16 | O75716 |
| BLC | C-X-C motif chemokine 13 | O43927 |
| Catalase | Catalase | P04040 |
| CNTF** | Ciliary Neurotrophic Factor | P26441 |
| IL-17 | Interleukin-17A | Q16552 |
| IL-17B | Interleukin-17B | Q9UHF5 |
| LEAP-1 | Hepcidin | P81172 |
| SDF-1 | Stromal cell-derived factor 1 | P48061 |
| TARC | C-C motif chemokine 17 | Q92583 |
| Vasoactive Intestinal Peptide | Vasoactive Intestinal Peptide | P01282 |
| CD40 ligand, soluble** | CD40 ligand | P29965 |
| dopa decarboxylase | Aromatic-L-amino-acid decarboxylase | P20711 |
| a1-Antitrypsin | Alpha-1-antitrypsin | P01009 |
| a2-HS-Glycoprotein** | Alpha-2-HS-glycoprotein | P02765 |
| Chitotriosidase-1 | Chitotriosidase-1 | Q13231 |
| CHL1 | Neural cell adhesion molecule L1-like protein | O00533 |
| CNDP1** | Beta-Ala-His dipeptidase | Q96KN2 |
| Endothelin-converting enzyme 1 | Endothelin-converting enzyme 1 | P42892 |
| HGFA** | Hepatocyte growth factor activator | Q04756 |
| MMEL2 | Membrane metallo-endopeptidase-like 1 | Q495T6 |
| RAP | alpha-2-macroglobulin receptor-associated protein | P30533 |
| SLAF5 | SLAM family member 5 | Q9UIB8 |
| VEGF sR2 | Vascular endothelial growth factor receptor 2 | P35968 |
| BMPER | BMP-binding endothelial regulator protein | Q8N8U9 |
| ALT | Alanine aminotransferase 1 | P24298 |
| BNP-32 | Brain natriuretic peptide 32 | P16860 |
| MMP-10 | Stromelysin-2 | P09238 |
| Carbonic anhydrase III | Carbonic anhydrase 3 | P07451 |
| CYTD | Cystatin-D | P28325 |
| Endocan | Endothelial cell-specific molecule 1 | Q9NQ30 |
| FGR | Tyrosine-protein kinase Fgr | P09769 |
| MATK | Megakaryocyte-associated tyrosine-protein kinase | P42679 |
| MK08 | Mitogen-activated protein kinase 8 | P45983 |
| PTK6 | Protein-tyrosine kinase 6 | Q13882 |
| AIP | AH receptor-interacting protein | O00170 |
| HSP 40 | DnaJ homolog subfamily B member 1 | P25685 |
| EF-1-beta | Elongation factor 1-beta | P24534 |
| eIF-5A-1 | Eukaryotic translation initiation factor 5A-1 | P63241 |
| LDH-H 1** | L-lactate dehydrogenase B chain | P07195 |
| Mesothelin | Mesothelin | Q13421 |
| sRAGE | Advanced glycosylation end product-specific receptor, soluble | Q15109 |
| C6** | Complement component C6 | P13671 |
| FGF-6** | Fibroblast growth factor 6 | P10767 |
| IL-17D | Interleukin-17D | Q8TAD2 |
| IL-6 sRa** | Interleukin-6 receptor subunit alpha | P08887 |
| IP-10 | C-X-C motif chemokine 10 | P02778 |
| Prekallikrein** | Plasma kallikrein | P03952 |
| MMP-2 | 72 kDa type IV collagenase | P08253 |
| 4EBP2** | Eukaryotic translation initiation factor 4E-binding protein 2 | Q13542 |
| EP15R | Epidermal growth factor receptor substrate 15-like 1 | Q9UBC2 |
| ERAB | 3-hydroxyacyl-CoA dehydrogenase type-2 | Q99714 |
| IF4G2 | Eukaryotic translation initiation factor 4 gamma 2 | P78344 |
| PA2G4 | Proliferation-associated protein 2G4 | Q9UQ80 |
| paraoxonase 1 | Serum paraoxonase/arylesterase 1 | P27169 |
| Spondin-1 | Spondin-1 | Q9HCB6 |
| Thymidine kinase | Thymidine kinase, cytosolic | P04183 |
| PSA | Prostate-specific antigen | P07288 |
| CRP | C-reactive protein | P02741 |
| FGF-16** | Fibroblast growth factor 16 | O43320 |
| ADAM12** | Disintegrin and metalloproteinase domain-containing protein 12 | O43184 |
| CHST2 | Carbohydrate sulfotransferase 2 | Q9Y4C5 |
| LRRT1 | Leucine-rich repeat transmembrane neuronal protein 1 | Q86UE6 |
| LRRT3 | Leucine-rich repeat transmembrane neuronal protein 3 | Q86VH5 |
| PDPK1 | 3-phosphoinositide-dependent protein kinase 1 | O15530 |
| TGM3 | Protein-glutamine gamma-glutamyltransferase E | Q08188 |
| ZAP70** | Tyrosine-protein kinase ZAP-70 | P43403 |
| MMP-12 | Macrophage metalloelastase | P39900 |
| FUT5 | Alpha-(1,3)-fucosyltransferase 5 | Q11128 |
| IL-34** | Interleukin-34 | Q6ZMJ4 |
| KIRR3 | Kin of IRRE-like protein 3 | Q8IZU9 |
| SLIK5 | SLIT and NTRK-like protein 5 | O94991 |
| Afamin | Afamin | P43652 |
| STRATIFIN | 14-3-3 protein sigma | P31947 |
| ACTH** | Corticotropin | P01189 |
| Caspase-2** | Caspase-2 | P42575 |
| Galectin-8 | Galectin-8 | O00214 |
| Integrin aVb5 | Integrin alpha-V: beta-5 complex | P06756, P18084 |
| LIF sR** | Leukemia inhibitory factor receptor | P42702 |
| MMP-1 | Interstitial collagenase | P03956 |
| ARTS1 | Endoplasmic reticulum aminopeptidase 1 | Q9NZ08 |
| cIAP-2 | Baculoviral IAP repeat-containing protein 3 | Q13489 |
| DBNL | Drebrin-like protein | Q9UJU6 |
| GPC5 | Glypican-5 | P78333 |
| GRN | Granulins | P28799 |
| GSTA3 | Glutathione S-transferase A3 | Q16772 |
| IF4A3 | Eukaryotic initiation factor 4A-III | P38919 |
| PARK7 | Protein DJ-1 | Q99497 |
| Tropomyosin 1 alpha chain | Tropomyosin alpha-1 chain | P09493 |
| TSG-6 | Tumor necrosis factor-inducible gene 6 protein | P98066 |
| CD226** | CD226 antigen | Q15762 |
| CRTAM | Cytotoxic and regulatory T-cell molecule | O95727 |
| EPHAA | Ephrin type-A receptor 10 | Q5JZY3 |
| GPNMB | Transmembrane glycoprotein NMB | Q14956 |
| IL-22BP | Interleukin-22 receptor subunit alpha-2 | Q969J5 |
| JAG1 | Protein jagged-1 | P78504 |
| JAML1 | Junctional adhesion molecule-like | Q86YT9 |
| LAG-3 | Lymphocyte activation gene 3 protein | P18627 |
| MO2R1 | Cell surface glycoprotein CD200 receptor 1 | Q8TD46 |
| Notch 1 | Neurogenic locus notch homolog protein 1 | P46531 |
| Semaphorin-6A | Semaphorin-6A | Q9H2E6 |
| SIG14 | Sialic acid-binding Ig-like lectin 14 | Q08ET2 |
| TCCR | Interleukin-27 receptor subunit alpha | Q6UWB1 |
| CK2-A1:B** | Casein kinase II 2-alpha:2-beta heterotetramer | P68400 P67870 |
| PDK1 | [Pyruvate dehydrogenase (acetyl-transferring)] kinase isozyme 1, mitochondrial | Q15118 |
| PCSK9 | Proprotein convertase subtilisin/kexin type 9 | Q8NBP7 |
| MP2K4** | Dual specificity mitogen-activated protein kinase kinase 4 | P45985 |
| ABL2 | Abelson tyrosine-protein kinase 2 | P42684 |
| SHC1** | SHC-transforming protein 1 | P29353 |
| Caspase-10 | Caspase-10 | Q92851 |
| Semaphorin 3E | Semaphorin-3E | O15041 |
| BAFF Receptor | Tumor necrosis factor receptor superfamily member 13C | Q96RJ3 |
| SLAF7 | SLAM family member 7 | Q9NQ25 |
| Stress-induced-phosphoprotein 1 | Stress-induced-phosphoprotein 1 | P31948 |
