# Supplementary material for: Proteomic analysis of heart failure hospitalization among patients with chronic kidney disease: The Heart and Soul Study
Source: PLoS One. 2018 Dec 17;13(12):e0208042. doi: 10.1371/journal.pone.0208042 (PMC6296511; doi:10.1371/journal.pone.0208042)
Supplement: S3 Table — (DOCX) [file pone.0208042.s004.docx]

**Supplemental Table 3. Protein – Heart Failure Associations with Interactions By CKD Status**

| **Target Full Name** | **Gene Name** | **CKD HR (95%CI)** | **NON-CKD HR (95% CI)** | **p-value** |
| --- | --- | --- | --- | --- |
| Spondin-1 | SPON1 | 1.11 (1.04, 1.19) | 2.16 (1.63, 2.86) | 1.4471E-06 |
| Inhibin beta A chain | INHBA | 0.82 (0.68, 0.99) | 1.40 (1.17, 1.68) | 8.58712E-05 |
| Protein jagged-1 | JAG1 | 1.07 (1.01, 1.15) | 2.49 (1.51, 4.11) | 0.000387186 |
| Follistatin-related protein 3 | FSTL3 | 1.08 (0.90, 1.29) | 1.94 (1.38, 2.71) | 0.001052666 |
| Insulin-like growth factor-binding protein 7 | IGFBP7 | 1.15 (1.06, 1.25) | 1.79 (1.42, 2.25) | 0.001205159 |
| Thrombospondin-2 | THBS2 | 1.19 (1.08, 1.32) | 1.84 (1.52, 2.24) | 0.001361616 |
| Delta-like protein 1 | DLL1 | 1.00 (0.90, 1.12) | 1.50 (1.20, 1.88) | 0.002234071 |
| Semaphorin-3E | SEMA3E | 1.01 (0.90, 1.12) | 1.53 (1.15, 2.04) | 0.00305364 |
| Tenascin | TNC | 1.19 (1.03, 1.37) | 1.81 (1.45, 2.28) | 0.003262116 |
| Cathepsin H | CTSH | 1.04 (0.87, 1.23) | 2.18 (1.26, 3.77) | 0.003653768 |
| T-lymphocyte activation antigen CD86 | CD86 | 0.98 (0.87, 1.10) | 5.53 (1.92, 15.93) | 0.004303147 |
| MHC class I polypeptide-related sequence A | MICA | 1.04 (0.98, 1.11) | 3.26 (1.50, 7.09) | 0.00432917 |
| Phospholipase A2, membrane associated | PLA2G2A | 1.13 (1.03, 1.23) | 1.69 (1.29, 2.20) | 0.004808789 |
| Estradiol 17-beta-dehydrogenase 1 | HSD17B1 | 0.96 (0.84, 1.10) | 1.31 (1.11, 1.54) | 0.006206126 |
| Serine/threonine-protein kinase 16 | STK16 | 1.05 (0.97, 1.12) | 1.69 (1.24, 2.30) | 0.007062764 |
| Neurogenic locus notch homolog protein 1 | NOTCH1 | 0.67 (0.56, 0.81) | 1.07 (0.82, 1.39) | 0.007156103 |
| Baculoviral IAP repeat-containing protein 7 Isoform beta | BIRC7 | 0.97 (0.78, 1.21) | 0.00 (0.00, 0.03) | 0.009331833 |
| Vascular cell adhesion protein 1 | VCAM1 | 1.10 (0.94, 1.28) | 1.60 (1.26, 2.04) | 0.010125206 |
| P-Selectin | SELP | 0.97 (0.84, 1.13) | 1.47 (1.13, 1.92) | 0.010819722 |
| Neuroligin-4, X-linked | NLGN4X | 1.03 (0.95, 1.13) | 1.31 (1.11, 1.55) | 0.011578124 |
| Neural cell adhesion molecule L1-like protein | CHL1 | 0.86 (0.71, 1.03) | 1.27 (0.98, 1.65) | 0.014911388 |
| Kallikrein-7 | KLK7 | 1.10 (1.02, 1.17) | 0.29 (0.08, 0.98) | 0.01661559 |
| Natural cytotoxicity triggering receptor 3 | NCR3 | 1.24 (0.83, 1.87) | 0.14 (0.02, 0.82) | 0.017548682 |
| Activated Protein C | PROC | 0.95 (0.83, 1.09) | 1.31 (1.06, 1.62) | 0.019208687 |
| Netrin receptor UNC5C | UNC5C | 0.95 (0.79, 1.15) | 1.37 (0.99, 1.89) | 0.021738312 |
| N-acetyl-D-glucosamine kinase | NAGK | 1.07 (0.91, 1.25) | 1.44 (1.14, 1.82) | 0.021798627 |
| Ectodysplasin-A, secreted form | EDA | 1.90 (1.12, 3.24) | 0.99 (0.73, 1.32) | 0.027436263 |
| 40S ribosomal protein SA | RPSA | 0.99 (0.84, 1.18) | 1.34 (1.12, 1.62) | 0.027656917 |
| SUMO-conjugating enzyme UBC9 | UBE2I | 1.24 (1.03, 1.51) | 0.81 (0.58, 1.13) | 0.028813865 |
| CD109 antigen | CD109 | 0.64 (0.34, 1.19) | 1.82 (0.79, 4.20) | 0.028937931 |
| Pappalysin-1 | PAPPA | 0.95 (0.82, 1.11) | 1.31 (0.96, 1.79) | 0.030046335 |
| Protein kinase C alpha type | PRKCA | 1.11 (1.00, 1.23) | 0.76 (0.52, 1.09) | 0.034174646 |
| Macrophage metalloelastase | MMP12 | 1.14 (0.98, 1.32) | 1.42 (1.14, 1.79) | 0.035909581 |
| DnaJ homolog subfamily B member 1 | DNAJB1 | 1.12 (0.97, 1.29) | 0.10 (0.01, 1.15) | 0.03869785 |
| Ribosome maturation protein SBDS | SBDS | 1.09 (0.98, 1.22) | 0.74 (0.50, 1.09) | 0.039042843 |
| Glypican-5 | GPC5 | 1.05 (0.97, 1.14) | 0.03 (0.00, 0.79) | 0.039232938 |
| Hepcidin | HAMP | 0.97 (0.83, 1.14) | 1.29 (1.00, 1.67) | 0.040151928 |
| 3-phosphoinositide-dependent protein kinase 1 | PDPK1 | 1.12 (1.03, 1.23) | 0.79 (0.55, 1.14) | 0.044862495 |
| Phosphatidylethanolamine-binding protein 1 | PEBP1 | 1.10 (0.92, 1.30) | 0.73 (0.49, 1.10) | 0.045072122 |
| Endostatin | COL18A1 | 1.09 (0.89, 1.33) | 1.53 (1.07, 2.19) | 0.045810565 |
| Neutrophil-activating peptide 2 | PPBP | 1.18 (1.03, 1.35) | 0.75 (0.49, 1.17) | 0.047345627 |
| Creatine kinase M-type:Creatine kinase B-type heterodimer | CKB CKM | 1.11 (0.90, 1.37) | 0.70 (0.42, 1.16) | 0.048878756 |
| Vascular endothelial growth factor receptor 3 | FLT4 | 1.40 (1.16, 1.69) | 1.04 (0.79, 1.37) | 0.049477213 |
